# Prophylactic use of interleukin 6 monoclonal antibody can reduce CRS response of CAR-T cell therapy

**DOI:** 10.3389/fmed.2023.1265835

**Published:** 2024-01-03

**Authors:** Baitao Dou, Shihui Ren, Ling Qiu, Xupai Zhang, Nan Zhang, Jiao Cai, Dan Chen, Qian Zhang, Hao Yao, Fangyi Fan

**Affiliations:** ^1^Department of Clinical Medicine, North Sichuan Medical College, Nanchong, Sichuan, China; ^2^General Hospital of the Chinese People’s Liberation Army Western Theater, Chengdu, Sichuan, China

**Keywords:** CAR-T cells, immunotherapy, interleukin 6 (IL-6), cytokine release syndrome (CRS), hematological malignancies (HM), prophylactic treatment

## Abstract

**Background:**

Chimeric antigen receptor T (CAR-T) cell immunotherapy is becoming one of the most promising treatments for hematological malignancies, however, complications such as cytokine release syndrome (CRS) seriously threaten the lives of patients. Interleukin 6(IL-6) monoclonal antibody is the common and useful treatment of CRS, however, it is not clear whether prophylactic use IL-6 monoclonal antibody before CAR-T therapy can reduce the incidence of CRS.

**Purpose:**

This study aims to systematically evaluate whether the prophylactic use of IL-6 monoclonal antibody can reduce the incidence of CRS.

**Data sources and methods:**

We searched the PubMed, Embase, web of Science, and Cochrane Library databases for studies that reported the prophylactic use of IL-6 monoclonal antibody in the treatment of CRS-related complications of CAR-T cell immunotherapy before December 2022. The literature is screened according to the established inclusion and exclusion criteria, relevant data are extracted, and the quality of the literature is evaluated using the scale Cochrane bias risk assessment tool, and the Review Manager 5.3 is used to draw for related charts. Since the two experimental data only provide the median, the maximum and minimum values of the data, the mean and standard (Standard Deviation, SD) are calculated by this document Delai, and finally use Review Manager for data processing, and STATA software for supplementation.

**Results:**

A total of 2 trials with a total of 37 participants were included in this study. Meta-analysis showed that compared with no use of IL-6 monoclonal antibody to prevent CRS, IL-6 monoclonal antibody was given to patients at 8 mg/kg one hour before CAR-T cell infusion, which reduced the incidence of CRS [RR: 0.41 95% confidence interval (0.20, 0.86) I[2] = 0.0% *P* = 0.338 *z* = −2.369 (*p* = 0.018)]. In subgroup analysis, compared with those who did not use IL-6 monoclonal antibody to prevent CRS, IL-6 monoclonal antibody was given to patients at 8 mg/kg one hour before CAR-T cell infusion, which reduced lactate dehydrogenase (LDH)[MD: −617.21, 95% confidence interval (−1104.41, −130.01) I[2] = 0% *P* = 0.88 *Z* = 2.48 (*P* = 0.01)], prophylactic use of IL-6 monoclonal antibody has a significant effect on reducing peak C-reactive protein (CRP) after CAR-T therapy [MD: −11.58, 95% confidence interval (−15.28, −7.88) I[2] = 0.0% *P* = 0.73 z = 6.14 (*p* < 0.00001)].

**Conclusion:**

The prophylactic use of IL-6 monoclonal antibody can significantly reduce the incidence of CRS complications after CAR-T therapy, can also reduce LDH vaule and peak CRP vaule after CAR-T therapy.

**Systematic review registration:**

https://www.crd.york.ac.uk/prospero/display_record.php?ID=CRD42023487662, identifier CRD42023487662.

## 1 Introduction

Chimeric antigen receptor T (CAR-T) therapy has been revolutionary in human cancer due to its potent and durable clinical responses (4). This kind of therapy is to modify the T cells of the patient *in vitro* with the specific CAR structure through various techniques, and then reinfuse them back into the patient’s body to better recognize and kill the patient’s tumor cells (5, 6). T cells are immune cells in the human body that can fight against abnormal cells including tumor cells (7). The CAR is mainly composed of four domains, namely, the outer domain for specific target antigen recognition, and the inner domain for providing co-stimulation and activation signals. These two domains are connected by a hinge and a transmembrane domain. Each domain plays an important role in killing tumor cells (8, 9). This therapy is currently approved by the FDA for the treatment of acute lymphoblastic leukemia (ALL), diffuse large B-cell lymphoma (DLBCL), follicular lymphoma (FL), mantle cell lymphoma (MCL), marginal zone lymphoma (MZL), and multiple myeloma (MM), improving the prognosis of patients with relapsed/refractory (R/R) hematologic malignancies (10–14). According to the retrospective SCHOLAR-1 study, the objective response rate (ORR) was 26% (complete response rate, 7%) to the next line of therapy for patients with refractory or relapse DLBCL. The median overall survival was 6.3 months (15). However, three CAR-T pivotal trials recruited heavily pre-treated patients, the majority of whom were chemorefractory (76% in ZUMA-1, 55% in JULIET, and 67% in TRANSCEND), proving that CAR-T cell therapy can improve ORR ranged from 52 to 74% (16). Thus, there is no doubt that CAR-T therapy for relapsed/refractory (R/R) hematologic malignancies is more than effective. This further demonstrates the effective role of CAR-T cell therapy in hematologic malignancies. At the same time, this therapy also has many limitations, such as antigen escape, targeted off-tumor effect, CAR-T related toxicity, etc., although there are more and more researches on improving this treatment limitation in the world, such as optimizing CAR. However, CAR-T related toxicity cannot be avoided (4, 7, 17, 18). The CAR-T related toxicities such as CRS, ICANS, and cytopenias threaten the lives of patients. Among them, CRS is the most common adverse reaction (19, 20). CRS is a systemic inflammatory response. The term “cytokine release syndrome” was first coined in the early

“90s,” when the anti-T-cell antibody muromonab-CD3 (OKT3) was introduced into the clinic as an immunosuppressive treatment for solid organ transplantation (21, 22). The mechanism by which CRS occurs is not yet fully understood. CRS can be induced by direct target cell lysis with consecutive release of cytokines such as interferon gamma (IFN-γ) or tumor necrosis factor alpha (TNF-α), or by activation of T cells due to therapeutic stimuli with subsequent cytokine release. These cytokines trigger a chain reaction by activating innate immune cells like macrophages and endothelial cells, which further release cytokines (22). IL-6, IL-10, and IFN-γ are among the core cytokines that are consistently found to be elevated in serum of patients with CRS (19, 22, 23). IL-6 is a four-helical protein of 184 amino acids. It has been confirmed that IL-6 *trans-*signaling is pro-inflammatory, whereas classic IL-6 signaling via the membrane-bound IL-6R is needed for regenerative or anti-inflammatory activities of the cytokine (24, 25). At the same time, many experiments have confirmed that IL-6 is the most important factor in CRS toxicity (26, 27). This is also the main difference between CRS and cytokine storm (CS) in mediators (27). CS can be caused by many factors, but CRS is mainly caused by the immune system. Meanwhile, CS seems to be more severe than CRS (28, 29). CRS can manifest in varying degrees of clinical symptoms in the skin, respiratory tract, heart, kidney, liver, coagulation, nervous system, and more. Mild patients may only experience cold-like symptoms and require only symptomatic treatment. However, severe patients may experience life-threatening conditions such as respiratory distress syndrome and myocarditis and require intensive medication treatment, including support from vasoactive vasopressors, mechanical ventilation, antiepileptic drugs, and antipyretics (30). Therefore, to better manage CRS patients, CRS management has undergone CTCAE version 4.03, Lee criteria, CTCAE v5.0, Memorial Sloan Kettering Cancer Center (MSKCC) criteria, Chiric Antigen Receiver Toxicity (CARTOX) criteria, Penn Criteria, American Society for Transplantation and Cellular Therapy (ASTCT) criteria (27, 30–32). Currently, the ASTCT hierarchical management is the most commonly used, CRS is defined as “any supraphysiological response following immunotherapy that results in the activation or involvement of endogenous or infused T cells and/or other immune effector cells.” Symptoms may be progressive and must include fever at onset, which may include low blood pressure, capillary leakage and end organ dysfunction (31). According to the severity of fever, hypertension and hypoxemia, the CRS grade can be divided into grades 1-4. At the same time, in the ASTCT hierarchical management fever is defined as temperature 38°C not attributable to any other cause. In patients who have CRS then receive antipyretic or anticytokine therapy such as tocilizumab or steroids (31). The frequency and severity of post-CRS varies by product (any grade CRS: 37–93%, grade 3/4 CRS: 1–23%) (11, 32–34). For low-grade CRS, management is mainly supportive, usually with intravenous fluids and antipyretics, and if more severe CRS develops, such as refractory hypotension or respiratory failure, will use drugs such as glucocorticoids. However, Research has confirmed that corticosteroids are very effective and inhibits the inflammatory response of CRS (35), but corticosteroids not included in CRS management because which would inhibit T cell function and may impair the persistence and antitumor activity of CAR-T cells. Compared with corticosteroids, IL-6 monoclonal antibody is the preferred initial treatment (30, 36). However, life-threatening CRS patients who do not respond to IL-6 monoclonal antibody targeted therapy should be reserved for high-dose corticosteroid therapy (37). In addition, different studies reported several biomarker, including LDH as a surrogate marker of hematologic tumor burden, CRP and Ferritin as general inflammatory markers, all three were associated with severe CRS (36, 38–41). Clinically, IL-6 monoclonal antibody are effective in the treatment of CRS, but whether preventive use in advance can reduce the incidence of CRS, to further reduce the pain caused to patients because of using mechanical ventilation and other methods in handling adverse reactions to CRS and avoid greater economic pressure on patients. We conducted a meta-analysis to evaluate the prophylactic use of IL-6 monoclonal antibody on the related complications of CRS after CAR-T efficacy.

## 2 Methods

### 2.1 Ethics

This article is based on previously conducted research and does not include research conducted by any of the authors with human participants or animals.

### 2.2 Literature search

The search was performed in the PubMed, Embase, web of Science, and Cochrane Library databases to collect literature on the curative effect of prophylactic use of IL-6 monoclonal antibody on CRS-related complications after CAR-T therapy. The retrieval strategy used a combination of medical subject headings (MeSH) and free words. The search terms included Cytokine release syndrome, Chimeric Antigen Receptor T Cell, Interleukin-6 antibody, Tocilizumab, sarilumab, satralizumab, siltuximab, olokizumab, clazakizumab, and prevention. The search time limit is from the establishment of the database to December 2022. The literature of the included studies was screened to find potentially eligible trials. In addition, we reviewed conference abstracts of unpublished work.

### 2.3 Eligibility criteria

#### 2.3.1 Inclusion criteria

Two researchers independently screened the literature according to pre-established inclusion and exclusion criteria. Inclusion criteria: (1) type of study: randomized controlled trial (RCT), whether blinded or not; (2) research objects: Patients diagnosed with hematological malignancies; (3) intervention measures: infusion of IL-6 monoclonal antibody one hour before CAR-T cell immunotherapy and no prophylaxis 1 h before CAR-T therapy; and (4) outcome indicators: the incidence of CRS was the main outcome indicator, the LDH value and peak CRP value were the secondary outcome indicators.

#### 2.3.2 Exclusion criteria

Research involving any of the following can be excluded: (1) animal experiments, cases, reviews, repetitions, and literature inconsistent with the research direction, and disagreements should be resolved through discussion; (2) literature with incomplete data and processed by data software, etc. Literature for which complete data were not available.

### 2.4 Data extraction and risk of bias assessment

Two investigators independently extracted data from the included trials. Differences are resolved through discussion. Refer to the data extraction guidelines for systematic reviews and Meta-analysis, and use pre-designed tables to extract data, including the first author, year of publication, research type, sample size, age, diagnosis of malignant blood diseases, interventions (intervention time, route of administration) and dose), control measures, and outcome indicators (see Tables 1, 2 for details). The risk of bias evaluation of the included studies was carried out in accordance with the Cochrane Systematic Review Handbook (42), and the evaluation items included: ① whether the randomization method is correct; ② implementation of allocation concealment; ③ implementation of blinding of participants and subjects; ④ blinding of outcome evaluation ⑤ completeness of outcome data; ⑥ selective reporting; and ⑦ other biases. each evaluation item was divided into high risk of bias, low risk of bias and unclear risk of bias (Figures 2, 3).

**TABLE 1:** Basic information of two studies.

References	Research type	Intervention time	Dose	Control measures	Outcome indicators
Caimi et al. (43)	Randomized controlled trial	Infusion of interleukin-6 monoclonal antibody one hour before CAR-T cell treatment	8 mg/Kg	No infusion of interleukin-6 monoclonal antibody one hour before CAR-T cell treatment	See Table 2
Ahmed et al. (44)	Randomized controlled trial	Infusion of interleukin-6 monoclonal antibody one hour before CAR-T cell treatment	8 mg/Kg	No infusion of interleukin-6 monoclonal antibody one hour before CAR-T cell treatment	See Table 2

**TABLE 2:** Outcome indicators of two studies.

		Paolo F Caimi, MD	Nausheen Ahmed MD
Experimental group	Number of events	6	1
	Total	15	6
	Median baseline LDH (U/L) (range)	184 (108–793)	299.2 (172–793)
	Baseline LDH, mean (U/L)	275.7227	392.7961
	Baseline LDH, SD (U/L)	196.9088	242.2844
	Peak CRP (mg/dl), median (range)	1.1 (0.2–22.3)	0.74 (0.18–22.29)
	Peak CRP, mean (mg/dl)	4.5934	6.0989
	Peak CRP, SD (mg/dl)	6.3528	8.6263
Control group	Number of events	6	6
	Total	8	8
	Median baseline LDH (U/L) (range)	263 (173–2954)	614.6 (173–2954)
	Median baseline LDH, mean (U/L)	857.0507	1048.0446
	Median baseline LDH, SD (U/L)	969.5299	969.5299
	Peak CRP (mg/dl), Median (range)	15.4 (12.1–23.7)	15.385 (12.15–23.7)
	Peak CRP, mean (mg/dl)	16.542	16.5452
	Peak CRP, SD (mg/dl)	4.0441	4.0266

### 2.5 Statistical analysis

All statistical analyzes were performed using Review Manager and STATA 14.0. For dichotomous variables, the relative risk (risk ratio RR) was used as the effect analysis statistic, and for continuous variables, the mean deviation (MD) was used as the effect analysis statistic, and each effect size provided its 95% confidence interval (CI). The heterogeneity among the included studies was analyzed by *Q*-test and *I*^2^, if *P* > 0.1 and *I^2^* < 50%, there was no heterogeneity, and a fixed effect model was used. On the contrary, if *P* < 0.1 and *I^2^* > 50% (heterogeneity is large), the random effects model is adopted, and subgroup analysis and sensitivity are used to detect the source of heterogeneity. Funnel plots to assess publication bias.

## 3 Results

### 3.1 Literature screening process and results

**FIGURE 1:**
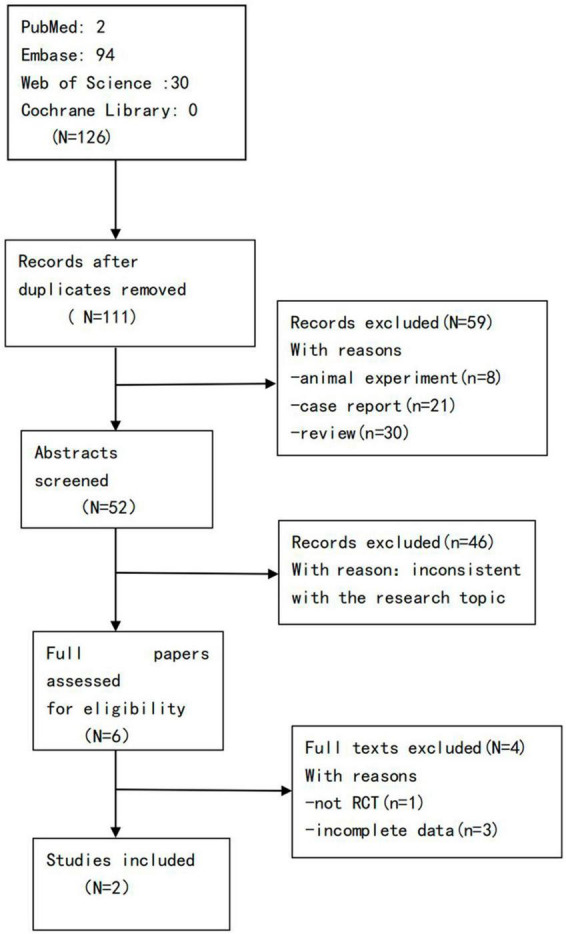
Flow diagram of the literature search strategy.

A total of 126 articles were obtained in the preliminary examination, including 0 articles from Cochrane Library, 2 articles from PubMed, 94 articles from Embase, and 30 articles from Web of Science, and a total of 111 articles after excluding duplicate articles. After reading the titles, abstracts, and full texts of the literature, according to the inclusion and exclusion criteria, 2 literatures were finally included, that is, 2 RCTs (43, 44) (Figure 1).

### 3.2 The basic characteristics of the included studies and the results of risk of bias assessment

**FIGURE 2:**
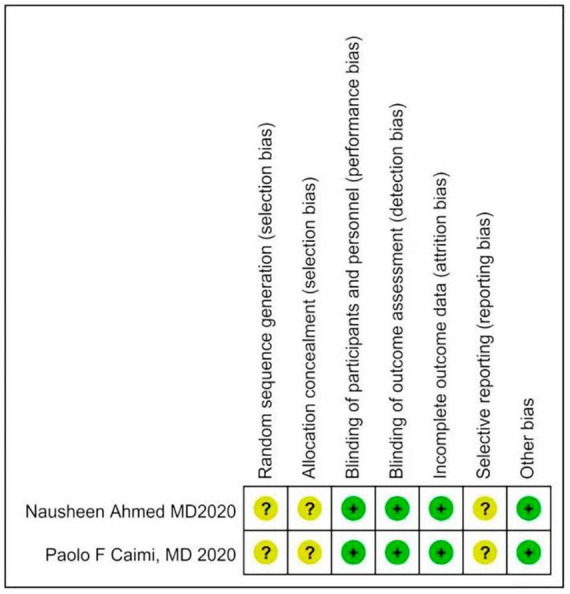
Risk of bias assessment through barplot.

**FIGURE 3:**
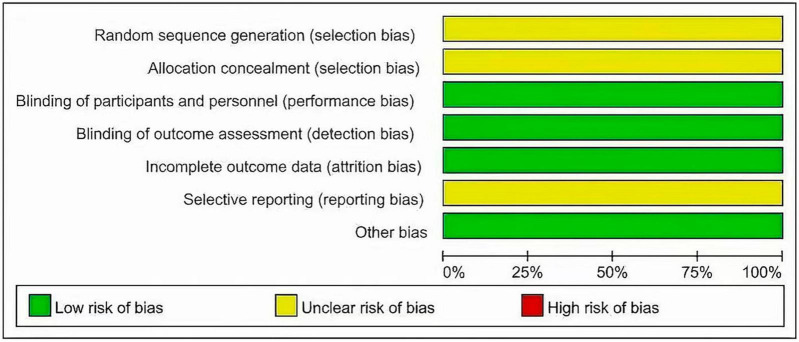
Risk of bias assessment through barplot.

The 2 articles that met the inclusion criteria included a total of 37 patients. The research objects of the 2 RCTs were DLBCL, Primary Mediastinal Large B Cell Lymphoma (PMBCL), FL, Transformed indolent, MCL, and (Burkitt lymphoma (BL). The basic characteristics of the included studies are shown in Table 1. The results of the risk of bias assessment of the included studies are shown in Figures 2, 3.

### 3.3 Meta-analysis

#### 3.3.1 The relationship between preventive medication and the incidence of CRS

**FIGURE 4:**
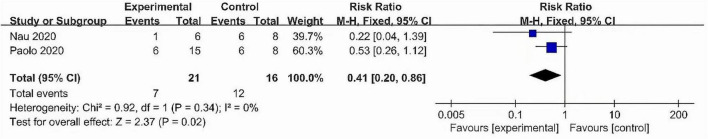
Forest plot of CRS incidence rate.

**FIGURE 5:**
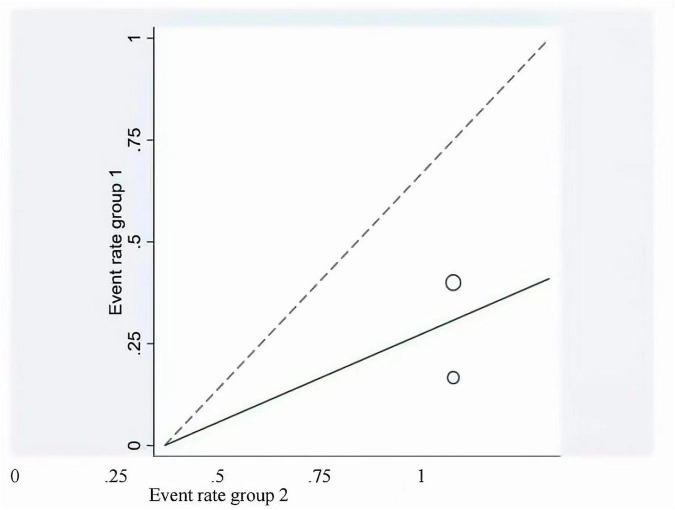
Labbe showing the article heterogeneity.

**FIGURE 6:**
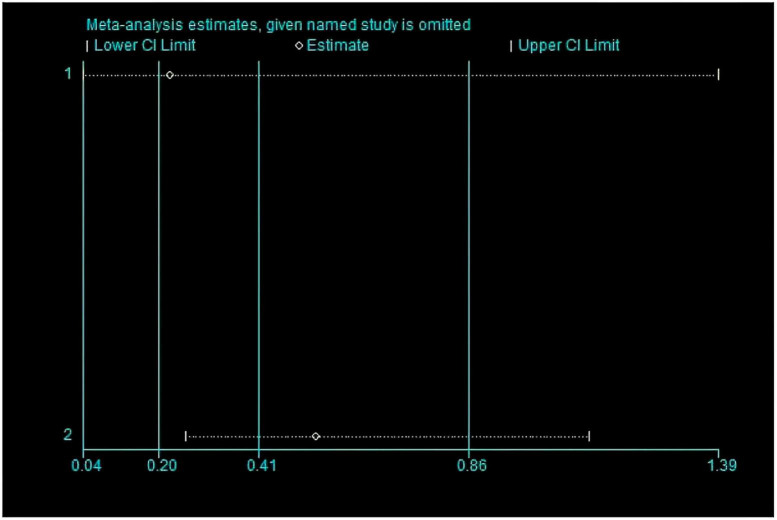
The picture use sensitive analysis to confirm whether have the heterogeneity.

**FIGURE 7:**
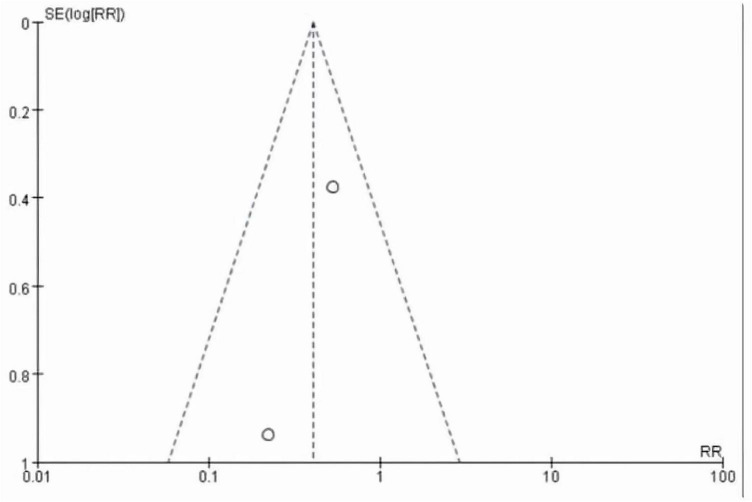
Funnel plot showing the publication bias in studies.

This meta-analysis included 2 studies with a total of 37 participants. The heterogeneity test of the incidence of CRS was performed by using the forest plot of the Review Manager (Figure 4). *I^2^* = 0.0% < 50% and the *Q*-test *P* = 0.34 > 0.1 indicated that there was no heterogeneity in the incidence of CRS, and at the same time, we further use of STATA to examine the L’ Abble plot (Figure 5) and sensitivity analysis (Figure 6) indicated that there was no heterogeneity in the occurrence of CRS, so the fixed effect model was used. The funnel plot (Figure 7) made by the Review Manage shows bilateral symmetry, so there is no publication bias. The combined analysis results (Figure 4) showed that compared with no use of interleukin-6 monoclonal antibody to prevent CRS, the RR value of the two studies was 0.41, and the 95% confidence interval was (0.20, 0.86), *z* = 2.37, *p* = 0.02 < 0.05, which is statistically significant, suggesting that interleukin-6 monoclonal antibody was administered to patients at 8 mg/kg one hour before CAR-T cell infusion for preventive medication, which reduced the incidence of CRS.

#### 3.3.2 LDH value of experimental group and control group

**FIGURE 8:**
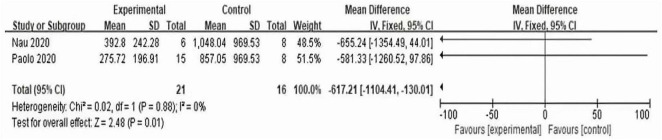
Forest plot of experimental and control LDH.

According to the forest plot (Figure 8), it can be seen that the LDH of the two trials is summarized, the MD value is −617.21, the 95% confidence interval is (−1104.41, 130.01), *Z* = 2.48, *P* = 0.01 < 0.05, which is statistically significant, It is suggested that the prophylactic use of interleukin-6 monoclonal antibody can reduce the LDH value after CAR-T therapy and reduce the tumor burden.

#### 3.3.3 Peak CRP value of experimental group and control group

**FIGURE 9:**
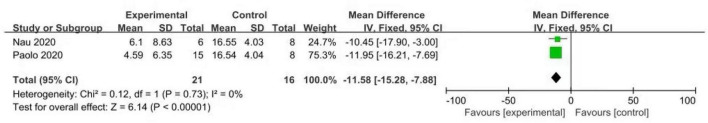
Forest plot of experimental and control peak CRP.

According to the forest plot (Figure 9), it can be seen that the peak CRP summary of the two trials, the MD value is –11.58, the 95% confidence interval is (–15.28, –7.88), *z* = 6.14, *p* < 0.00001, there is statistical significance, suggesting that the prophylactic use of IL-6 monoclonal antibodies can reduce the peak CRP after CAR-T cell therapy and relieve the symptoms of CRS.

## 4 Discussion

Through this meta-analysis, we found that prophylactic administration of IL-6 monoclonal antibody at 8 mg/kg 1 h before CAR-T infusion reduced the incidence of CRS. With the advancement of related technologies including genetic engineering and immunotherapy, chimeric antigen receptors (CARs) have gone through five generations (45). CAR-T therapy has brought hope to patients with hematological malignancies, but the severe side effects of which have reduced the benefits of patients. How to reduce or avoid the side effects of this therapy is a hot topic in research all over the world. Researchers have confirmed that the construction of CAR structure, the introduction of suicide genes into CAR-T cells, the regulation of cytokine activity in macrophages, and the autonomous neutralization of key cytokines can alleviate the symptoms of CRS (46). At the same time, combined therapy of CAR-T with programmed death 1 (PD-1)/programmed cell death-ligand 1 (PD-L1), Ibrutinib, PI3K inhibitors and other drugs can enhance the function of CAR-T cells and reduced cytotoxicity (45). IL-6 is a cytokine that is involved in the occurrence of CRS. IL-6 monoclonal antibody binds to IL-6, inactivates IL-6 cytokine, and alleviates the symptoms of CRS. Previous studies only showed IL-6 monoclonal antibody were started when patients show evidence of CRS grade 2 or higher, and the regulatory practice for patients in this meta-analysis was changed to 8 mg/kg BW one hour before CAR-T cell infusion preventive medication to reduce the side effects of this therapy (43, 44). One study showed that 6/8 (75%) of patients who did not receive preventive treatment observed any level of CRS, while 1/6 (17%) of patients who received preventive treatment observed CRS (*p* = 0.05). At the same time, this study also showed a statistically significant difference in CRP peak, with a CRP peak of 0.74 mg/dl in patients receiving preventive treatment and 15.3 mg/dl in patients not receiving preventive treatment (44). Another study suggests that CRS of any grade was observed in 6/8 (75%) of patients without prophylactic tocilizumab vs. 6/15 (40%) in patients treated with prophylactic tocilizumab (*p* = 0.23), whereas CRS grade > 1 was observed in 5 patients (62.5%) without prophylactic tocilizumab and in 3 patients (20%) treated with prophylactic tocilizumab (*p* = 0.02) (43). Finally, Our analysis also confirmed that this method can reduce the incidence of CRS. At the same time, based on the conclusions of these two articles, we can conclude that preventive use of Interleukin 6 monoclonal antibody can reduce the occurrence of CRS at any level. Unfortunately, the two studies adopted did not have specific data on the classification of CRS levels in patients who were prevented from using Interleukin 6 monoclonal antibody, so more experiments are needed to further clarify this. Meanwhile, higher cytokine levels and elevated inflammatory markers, such as ferritin, CRP, IL-6, Interferon gamma (IFN-γ), and tumor necrosis factor-a may reflect the severity of CRS (47). This study also has been able to confirm that IL-6 monoclonal antibody administered to patients at 8 mg/kg one hour before CAR-T cell infusion can reduce peak CRP inflammatory indicators. Meanwhile, regarding IL-6 cytokines, an article adopted suggests that although the plasma concentration of IL-6 increase, preventive use of IL-6 monoclonal antibodies before infusion of CAR-T cells can still reduce the incidence of severe CRS (43). However, due to the small sample size in this experiment, there is still a need for extensive experimental research to further elucidate the relationship between IL-6 cytokine concentration and the incidence of CRS. As mentioned above, corticosteroids are effective in the treatment of CRS clinically, but whether preventive medication can reduce the incidence of CRS remains to be further studied.

This study also had limitations. First, it is difficult to rule out publication bias because our meta-analysis included only 2 studies. Furthermore, since the two experimental data only provided the median, the maximum and minimum values of the data, the mean and SD values were obtained by the calculation methods in this literature (1–3).

In general, through this meta-analysis, we found that IL-6 monoclonal antibody administered to patients at 8 mg/kg one hour before CAR-T cell infusion reduced the incidence of CRS. Although the number of included samples is small and a large amount of data is needed to confirm, it provides a direction for clinical work to reduce the CRS side effects of CAR-T cell immunotherapy.

## Data availability statement

The raw data supporting the conclusions of this article will be made available by the authors, without undue reservation.

## Author contribution

BD: Data curation, Formal analysis, Methodology, Writing – original draft. SR: Formal analysis, Methodology, Writing – original draft, Investigation, Project administration. LQ: Formal analysis, Software, Writing – review and editing. XZ: Writing – review and editing, Investigation, Project administration. NZ: Project administration, Writing – review and editing, Validation. JC: Validation, Writing – review and editing, Investigation. DC: Writing – review and editing, Funding acquisition, Supervision. QZ: Writing – review and editing, Validation. HY: Writing – review and editing, Funding acquisition, Supervision. FF: Conceptualization, Funding acquisition, Supervision, Validation, Visualization, Writing – review and editing.
